# Coulometer from a Digitally Controlled Galvanostat with Photometric Endpoint Detection

**DOI:** 10.3390/s22197541

**Published:** 2022-10-05

**Authors:** Domingo González-Arjona, Emilio Roldán González, Germán López-Pérez, Manuel María Domínguez Pérez, Marina Calero-Castillo

**Affiliations:** Department of Physical Chemistry, Faculty of Chemistry, University of Sevilla, 41012 Sevilla, Spain

**Keywords:** electrochemical instrumentation, galvanostat, coulometry, optical detector

## Abstract

In this work, a coulometer was developed from a digitally controlled galvanostat. A simple colorimeter based on a RGB LED was used as a light emitter coupled to light detectors, while light dependent resistance (LDR) and photodiodes have been developed as endpoint detectors. Both hardware and software have been adapted from the original galvanostat design. Regarding the hardware, new electrical signal conditioners (filters and voltage dividers) were included to optimize the working system. The software was developed based on an open source Arduino UNO microcontroller. The different variables that control the titration process are managed by an add-in module for Excel data acquisition software that is freely available. A study of the possible variables that influence the titration process has been carried out. The system was tested with two classical coulometric titrations such as iodometry (thiosulfate, ascorbic acid) and acid/base (potassium acid phthalate as standard). The developed system is versatile as different endpoint color indicators can be employed (starch and phenolphthalein for the investigated reactions). Different experimental arrangements have been studied: the nature of the electrodes (Pt, Ag), type of cells (two separate compartments or a single compartment), and light detectors (LDR, photodiode). The influence of several experimental parameters (both electrical, light, and integration time) was studied and chosen to obtain the best performance of the complete system. Reproducibility results below 1% can be obtained under controlled conditions. In the case of acid/base titrations, the presence of atmospheric carbon dioxide was detected, whose interference was mainly affected by the stirring rate and the titration time.

## 1. Introduction

One of the most common analytical techniques of quantification in a chemical laboratory is volumetric titration. The unknown concentration of a compound in a sample is obtained by adding an amount of reactant that has a fast, irreversible, and quantitative reaction with it. The amount of reactant added is stopped when the compound under study in the sample is consumed. Thus, a reliable method for detecting the endpoint needs to be provided. Moreover, the concentration of the reactant solution to be added has to be known precisely and its concentration kept stable, among other analytical considerations.

Electrochemistry, through electrolysis techniques, can be employed for the controlled production of numerous reagents in situ, which can be used as titrant agents. This is an especially adequate methodology for the case of unstable or difficult to manipulate reactants due to particular reaction conditions [1,2]. Measurements of the electric current can be performed with great precision, allowing for accurate determination at very low concentration levels. In coulometric methods based on Faraday’s law, the number of coulombs that passed through the cell are quantitatively related to the amount of compound electrolyzed. The charge associated with the electrons is used as the primary standard reagent, generating the titrant in controlled quantities within the electrolytic cell, which avoids the necessity of preparation and the storage reagent standard solutions. In any case, the electrochemical reaction has to be a single and fast process, with a well-defined stoichiometry.

The first quantitative application of electric charge was performed at the beginning of the 20th century, but it was not until 1938 that a succession of papers appeared with the term “coulometric” in the title [3]. These papers, together with the advent of electronic instrumentation and the numerous and assorted electro-generated chemicals, led to the widespread application of the coulometric analysis. A. J. Bard published an extensively review on coulometric analysis [4] and together with chapters 11 and 15 in his popular textbook, both constitute an excellent starting point for study and a rich source of references [5]. The coulometric technique shows some important advantages over the classical titration methods such as high sensitivity, the usage of aa very low amount of reagent required, and/or unstable compounds as titration agents, no primary standard is needed and the main reactant (electrons) can be precisely controlled. Nevertheless, some drawbacks that can be overcome are also present such as a more complex instrumentation, the non-commercial availability of specific experimental devices, specific training for the laboratory specialist, and normally, a higher budget hardware [6,7,8].

A literature review from 2000 to the present on the aspects of the “coulometry at constant current” was carried out. Around thirty related articles were referenced as an indication of the usefulness of the constant-current coulometry. In the last six years, some articles based on the use of coulometry, from the analytical point of view, were published describing the application to the detection and quantification of a variety of compounds from metals, pharmaceuticals, water, antioxidants, gases, and certified materials [9,10,11,12,13,14,15,16,17,18]. Furthermore, numerous papers on coulometry have been published in education journals due to their instructional character (see recent papers on coulometry for chemical education [19,20,21] and the references therein).

Two methods can be distinguished based on the electrical variable controlled during the electrolytic process, with either the electrical potential or current being kept constant. In constant-potential electrolysis, the redox reaction of interest is controlled by the potential value, avoiding other possible electrochemical side reactions. When no compound is left to be consumed by the electrolysis, the process ceases. Thus, no separate signaling/endpoint detection method is necessary, as the current decreases exponentially to a zero value. The time for complete reaction is large and it is usually difficult to discern the endpoint from the residual current [5]. Moreover, the instrumentation is more complicated, more expensive, and can be applied to a limited number of substances [22]. On the other hand, constant-current electrolysis allows for a greater number of substances to be converted and it provides a straightforward relationship between the electric charge passed and the electrolysis time. Thus, the electrolysis time is directly proportional to the amount of compound generated by the redox reaction. Moreover, the necessary electronic instrumentation is simpler (a galvanostat). Thus, at constant current and when the redox reaction of interest reaches completion, the galvanostat modifies the applied potential to keep the current constant, which inevitably leads to side reactions. Therefore, a system indicating the end of the electrolysis time for the studied redox process is necessary.

In any case, for analytical purposes, a 100% current efficiency is necessary with both methodologies. One way of providing an effective means of control, minimizing the side reactions, is by monitoring the evolution of the electrical variable that is not kept constant during the electrolysis. Thus, exponential changes in the evolution of the current when operating in potentiostatic mode or sudden changes in the potential in galvanostatic mode can be indicative for side reactions, which lower the process efficiency. An additional complication in designing an electrolysis cell arises when the chemical components formed at both electrodes can react each other in the bulk solution. In this case, the solution around each electrode has to be separated by an electrically conducting membrane.

The coulometric system developed in this project was based on an adaptation of the original prototype for a digital galvanostat [23], used in the characterization of primary and secondary batteries. Initially, the same digital microcontroller (Arduino UNO) [24] was utilized due to its low cost, user-friendly programming environment, and open software–hardware environment that runs on Windows, Mac OsX, and Linux operating systems [25]. The input/output data by serial communication were detected/processed by using the free Data Acquisition tool PLX-DAQ [26] embedded as a controller in an Excel type spreadsheet, as previously described [27]. Thus, the input parameters for the instrument control and the data acquired can be performed via spreadsheet, avoiding the need to upload the software to the microcontroller every time an experimental run is started. Moreover, the range of different input parameters is validated in the spreadsheet, while the signal of the optical detector is also graphically monitored during the initial electrolysis process.

Nevertheless, some adaptations of the original galvanostat hardware/software are required to perform coulometric titrations. Thus, the battery connectors are now electrical terminals for the electrolysis cell. The terminal used to read the battery potential during its characterization is now employed to monitor the status of the electrolysis cell for the detection of incorrect electrode connections, the potential obstruction of the anode/cathode separation membrane, and even for recording possible changes that may be due to the existence of side reactions.

Different electrochemical techniques can be used as endpoint detectors. These are based on checking the abrupt change of an electrical variable at the endpoint. An additional electric circuit with a pair of electrodes isolated from the electrolytic generator system is used: potentiometry (electric potential against a reference electrode), amperometry, and dead-stop [28]. The use of an independent electric circuit, combined with the microcontroller’s difficulty in reading directly negative values of voltages, complicates the endpoint detector design.

On one hand, the spectrometric detectors are based on a significant change in color (UV–Vis), either by the addition of an indicator or by the color change in the electrolytic solution itself. Notwithstanding its versatility, the implementation of the spectrophotometric technique is generally more expensive. A possible way around such budgetary constraints resulting from using spectrometry without a big loss in flexibility can be resolved by the use of a colorimeter for the visible detection [10,20,25]. In this work, a simple colorimeter was developed based on a RGB LED as a light emitter, coupled to a visible light detector as a photo resistor (LDR/photodiode).

The complete system was tested for classical iodometric and acid/base titrations (thiosulfate, ascorbic acid, perchloric acid, and potassium hydrogen phthalate). Starch and phenolphthalein were used as the visible endpoint indicators, respectively. Multiple arrangements of electrode materials (Pt, Ag), electrolytic cells (two separated half-cells or a single compartment), light detectors (LDR, photodiode), and several combinations of experimental parameters (both electrical, light and integration time) have been explored to obtain the best performance from the developed coulometric system. Some important advantages can be achieved with the proposed design. On one hand, the usage of a microcontroller allows for very precise control of the electrolysis time, and on the other hand, the open source system reduces the hardware budget required for the titration device.

The versatility of the system allows for the development of other types of applications using galvanostatic methods [3], for example, the measurement of the thickness of metallic coatings. The proposed system allows the potential difference applied between the electrodes to be monitored simultaneously with the galvanostatic process. An appreciable change in the potential value will indicate that the electrolysis of the metallic coating has been completed. Therefore, taking into account Faraday’s laws, the density of the metal and the surface area exposed, the elapsed time will be proportional to the thickness of the metal coating. This would require a complete change in the design of the galvanostatic cell, the software, and the study of the possible variables involved.

## 2. Materials and Methods

### 2.1. Hardware

(a) Galvanostat: The coulometer hardware setup mainly constitutes the adaptation of the original galvanostat prototype [23,27], a flexible optical endpoint detection system, and an Arduino UNO employed as a microcontroller for handling the entire system.

The original galvanostat design was based on a scale/inverter operational amplifier. The current ranges were manually selected (jumpers) by a combination of the resistors. Switching on/off of the galvanostat operation was digitally controlled. A differential operational amplifier (OA) was used to read the battery’s actual voltage. The actual current value within the full scale selected was controlled by applying a DC bias voltage. This DC potential was digitally generated by using a pulse width modulated (PWM) signal [29], which was previously passed through an active second order 1 Hz low-pass filter. Thus, 256 different current values could be generated for the full scale selected. The polarity of the current could be controlled digitally by activating or deactivating its passage through a voltage inverter. The schematic design of the original galvanostat prototype can be found in the Supplementary Materials in [23].

Thus, the adaptation of the galvanostat described above mainly consisted of the substitution of the BAT connections for the electrodes of the electrolysis cell. The other digital controls were kept to adjust the current passing through the electrolysis cell. With this configuration, the selected current, which is passed through the electrolytic cell, was kept constant by continuously adjusting the voltage applied to the battery under study. Thus, if the electric resistance in the electrolytic cell increases during the process, to maintain the current value, the applied voltage will also increase. Moreover, in the case that during the setup of the electrolytic cell, the resistance between the electrodes is abnormally high, for example, due to a connection failure, the voltage measured by the differential OA will increase to its maximum value of 15 V (the value of the power source). Therefore, by monitoring this voltage during the electrolysis process, it is possible to detect changes in the efficiency of the electrolysis process and/or a failure in the electrical connection between the electrodes. In any case, this voltage should be a positive value and it has to be scaled for the adequate operation of the microcontroller. The correct polarity is imposed by using the digital control of charging/discharging, ensuring that a positive voltage is obtained. To scale this electrical signal to the standard value of 5 V (the maximum value supported by the microcontroller), a simple voltage divider was used, guaranteeing a maximum output voltage of 4.7 V for a 15 V power source. The voltage divider output was read by one analog input in the microcontroller.

**Figure 1:**
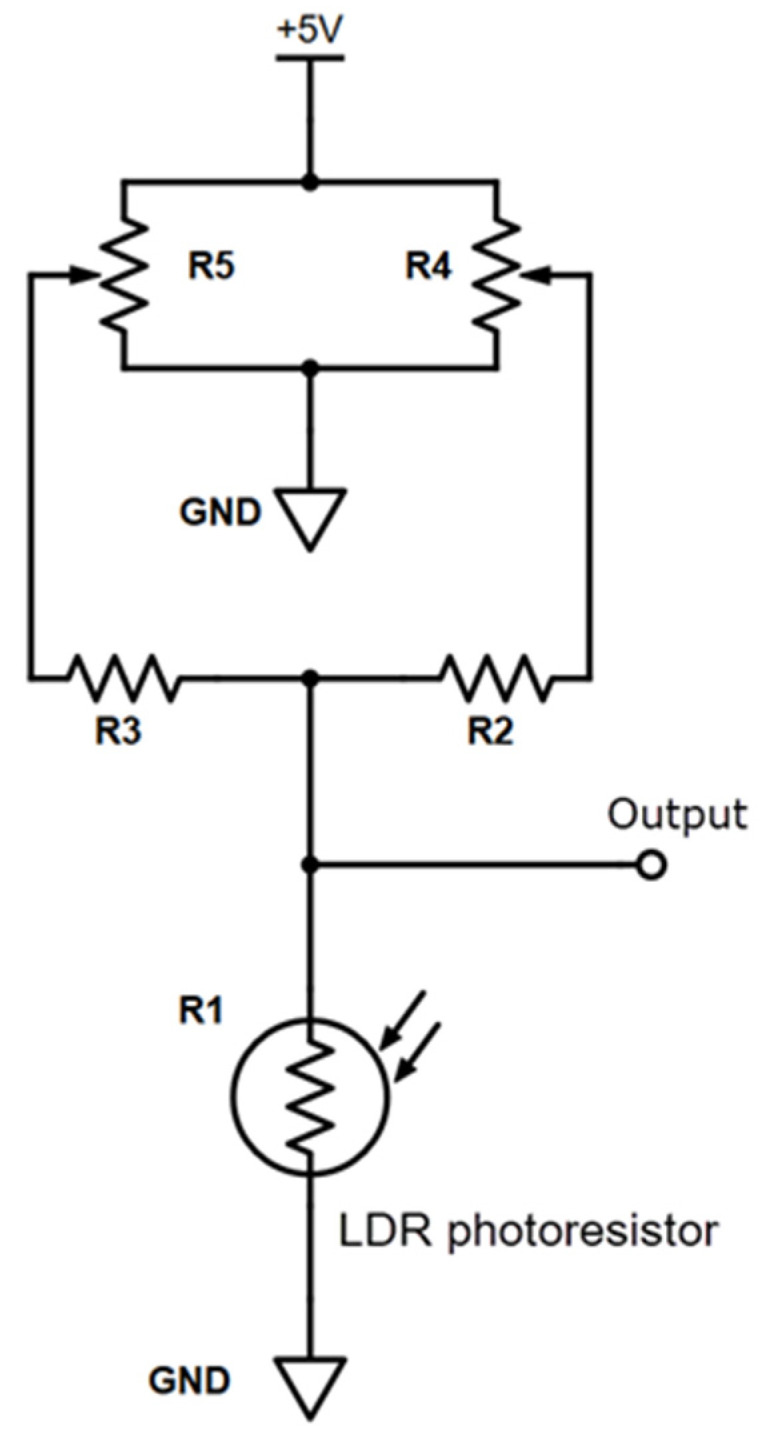
A schematic of the voltage divider LDR connection. R1: LDR (photo-resistor); R2: 100 kΩ, R3: 1 MΩ, R4 and R5: 10 kΩ multi-turn for the rough and fine-tuned voltage output adjustment, respectively.

(b) Electrolysis cell: Electrodes were placed in a standard 150 mL beaker. A platinum coil with an area of 1.25 cm^2^ was employed as the indicator electrode. A silver square sheet with an area of 3 cm^2^ was employed as a counter electrode for single compartment cell experiments. A cylindrical tube (15 mm diameter) with a fritted glass bottom covered with agar–agar gel saturated with KCl was used as a salt bridge when working with a two-compartment electrolysis cell. In the latter case, another Pt coil was used as the counter electrode.

(c) Endpoint detector system: A simple colorimeter was developed based on a RGB LED light source [30], coupled with two kinds of light detectors: a photo resistor LDR [31] and a photodiode OPT101 [32]. The endpoint detector system allows for a limited selection of wavelengths, centered close to the three maxima for the RGB light (460 nm for blue, 520 nm for green, and 630 nm for red) to obtain the maximum sensitivity depending on the color developed in the solution. Better sensitivity is achieved when the color for the RGB illumination and the indicator color are nearly complementary. The intensity of the light emitter color is controlled by using three microcontroller PWM digital pins. In our case, a single color of the RGB LED was turned on, taking into account the color emitted by the chemical indicator used. The electrical signal activating the emitter can be set easily, and the change in the light intensity reaching the detector has to be scaled and adjusted to maximize the voltage change.

(d) LDR detector configuration: A LDR changes the resistance from tens of kΩ under bright light (1000 Lux) to approximately 1 MΩ in the dark (<0.1 Lux). They are readily available at low cost but have a drawback because its electrical response is slow and not linear. Nevertheless, cheap LDRs possess sufficient sensitivity and can be easily implemented. The photo conductive cell is electronically arranged in a voltage divider configuration in which the initial output value can be precisely selected by using a couple of multiturn potentiometers: one for rough selection and another for fine-tuning the output voltage adjustment. The output voltage decreases as light intensity increases nonlinearly. Figure 1 schematically shows the connections according to [33].

By using this circuit and employing two ten-turn potentiometers, the output voltage can be adjusted between 0 V and +5 V with a resolution (R4, R5) of about one millivolt. This provides great versatility for the selection of the response range of the photoresistor element.

**Figure 2:**
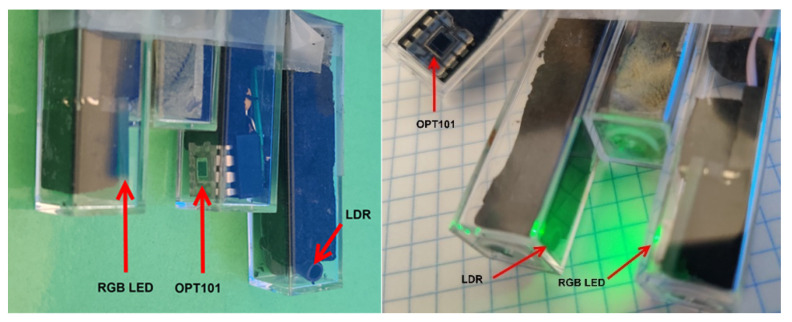
Colorimetric endpoint detector ensemble (LDR/OPT101_RGB_LED) showing the emitter, the light detectors, and the spatial arrangement.

(e) Photodiode (OPT101) detector configuration: Light detector devices and electronic ensembles of greater sensitivity, which exhibit linear responses, can be employed, for example, those based on photodiodes, phototransistors, and even a simple LED [20]. Based on the adequacy of its characteristics for this application, a photodiode (type OPT101) was selected [32]. Basic connections for this device were performed following the schematics depicted in Figure 2 in [32] minimizing the number of electronic components required. Minimizing the high-frequency noise that normally occurs in data acquisition when interfacing the digital microcontroller to an analog light detector or sensor is imperative. For both detectors, a simple RC low pass filter with a cutoff frequency of 15Hz was inserted between the detector output and the analog input of the Arduino [28].

(f) Colorimetric detector arrangement: The emitter and detector were placed at the bottom of two standard plastic spectrophotometric cuvettes (1 cm of path light). Both electronic devices were kept in place using a non-conducting black foam. The emitter and the detector cuvettes were fixed facing one another and separated by a third one, raised with respect to the other two, and kept in place with glue. The experimental assembly is depicted in Figure 2.

In this way, the colorimeter detector assembly can be easily immersed in the cell solution, keeping the distance and position of both the detector and emitter fixed. When the solution under analysis started to develop a change in color due to the excess electrochemically-generated reactant interacting with the chemical indicator, the intensity of light reaching the light sensor decreased and the output voltage changed. When the LDR detector was employed, the output voltage increased, while it decreased when the photodiode (OPT101) was used. The electrolysis process continued until the output voltage reached a preset value. The color of the solution was kept uniform by using a magnetic stirrer (Stuart CB162).

### 2.2. Software

The galvanostat was controlled by an Arduino UNO microcontroller, whose description and calibration is described in the literature [23,27,34]. However, the development of the chrono-coulometry experiments not only required the aforementioned hardware adaptation, but also the implementation of software adapted to this type of experiment. In order to perform a proper coulometer operation, the software has to be uploaded into the microcontroller and, in addition, an Excel sheet in which the add-in is embedded has to be used [26,27]. A simplified description of the operating software of the coulometer will be given in the following development, while further details are given in the Supplementary Materials.

Three fundamental stages can be distinguished within the program, according to the general scheme of the microcontroller: the initialization, setup, and loop procedures. The first stage corresponds to the initialization and assignment of the different pins of the microcontroller. In this same stage, the types of variables that will be used in the experiment are defined: accumulated time, potential, and current intensity. The corresponding values of the ordinate and slope corresponding to the linear calibration were also set.

The next step, which is also performed only once, initially configures the default values of the variables and establishes the serial port communication. The resistance between the electrodes is checked to verify that its value is low. Thus, the galvanostat is momentarily switched ON using a very low current value. Simultaneously, the voltage developed between the electrodes is read, and if the value is higher than the one previously specified, the program ends, indicating that there is an error in the electrical connection. The experimental variables are then read from the Excel sheet using the PLX_DAQ serial communication add-on and are checked for compliance with the appropriate ranges set previously. Thus, the current input value entered in the spreadsheet has to be a value within the selected sensitivity range manually selected using the PCB prototype galvanostat [23]. The process of verification ends when all requirements for the variables are satisfied: sensitivity, actual current value, RGB LED brightness, and timing parameters.

In coulometry, it is common to perform a pre-titration to select the endpoint value. Thus, a small amount of the sample is added to the electrolyte solution and titrated to a certain detector value. Subsequently, when the sample is added, the titration will end at the previously selected endpoint [35]. Thus, this preset value for the optical detector corresponding to the endpoint is then set.

Previously, the color of the light emitter and the most appropriate intensity would have been selected. The actual detector level value, given by the detector, is verified before the titration can be performed. Thus, for the LDR detector, the actual value should be lower than the endpoint selected, and the opposite for the photodiode OPT101. The light detector type has to be previously selected, in order to choose the adequate program code lines (see Supplementary Materials for details). Setting the experimental parameters concludes when there are no error messages on the detector level and required parameters. Two check marks of these possible errors have to be erased on the controller. Finally, the status of the start/stop switch is checked and its position is held until the start of the titration.

As the communication between the Arduino and the spreadsheet is performed via RS232 serial, some parameter synchronization errors may occur, but this can be easily solved by resetting the Excel controller without losing any data.

**Figure 3:**
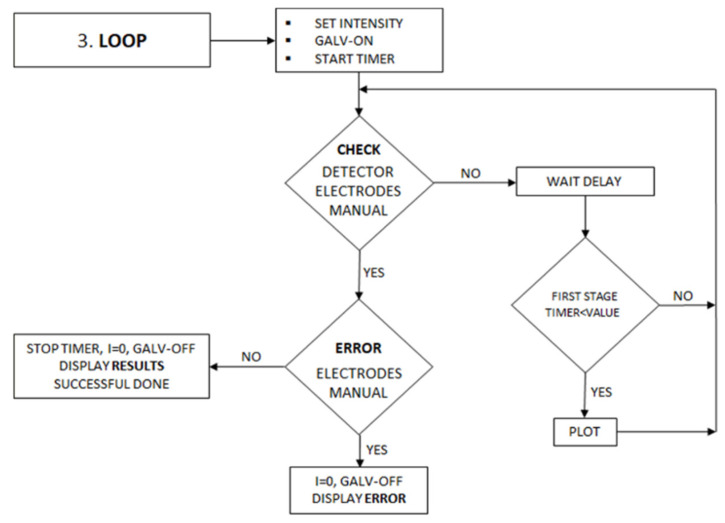
Flow diagram for the measurement and endpoint control. The error conditions are also verified: high resistance and emergency stop.

In the third part of the program, the process of measurement, endpoint control, and the estimation of results takes place. Figure 3 shows a flow diagram for these processes.

The sample volume is then added to the solution and the process is initiated by activating the start/stop switch. Electrolysis at a given intensity takes place until the color of the solution reaches the previously selected detector value. During the first electrolysis process, the detector value is plotted on an Excel graph versus time, allowing it to be monitored. When the selected detector value is reached for the first time, the electrolysis is stopped for a certain period, fixed as one of the initial conditions. This procedure allows the color in the solution to become uniform. Next, the electrolysis is resumed and if this next electrolysis time is shorter than the value specified under the initial conditions, the titration is considered completed and the total electrolysis time is determined. Completion of the titration in stages minimizes the error due to instantaneous coloration exceeding the preset value of the detector. The number of stages is limited to ten cycles.

In the second and subsequent electrolysis reactivation processes (stages), communication through the interface with Excel is inhibited, stopping the simultaneous plotting of the graph for a precise control of the elapsed time. Throughout the electrolysis process, the program monitors the value of the applied potential to detect possible increases in electrical resistance between the electrodes. If this situation occurs, the process stops, showing an error message. The manual stop switch is also continuously monitored, terminating the entire process when activated.

**Figure 4:**
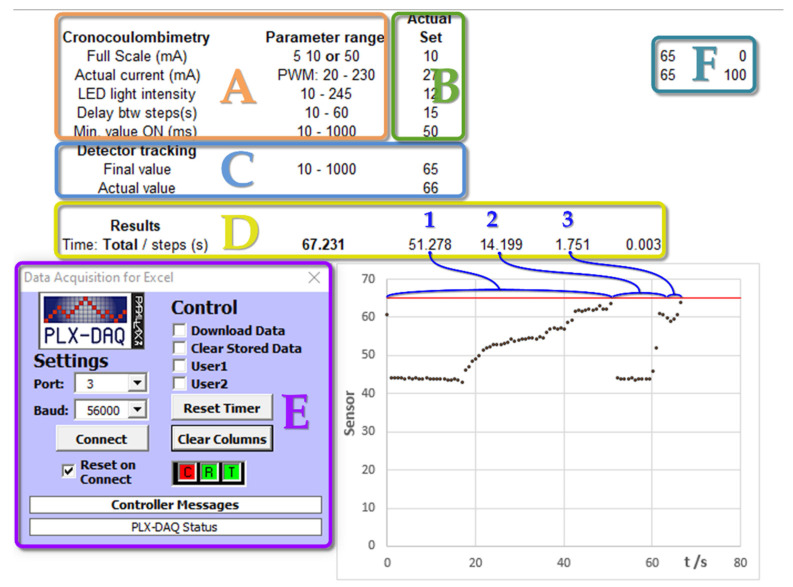
A screenshot of the spreadsheet interface for testing the entire coulometric titration program with a dummy cell and using the LDR as a light sensor. (**A**) Input parameters and verification ranges. (**B**) Actual value set. (**C**) Detector levels set. (**D**) Measurement results: numbers 1, 2, 3 refers to different experimental stages. (**E**) Add-on module for serial communication. (**F**) Plot scales.

Figure 4 shows a screenshot of the spreadsheet interface for a simulated measurement process using LDR as the light detector and a resistor as the dummy electrochemical cell. Initially, the parameters to be used in the experiment are set in section B, as indicated in Figure 4. These are checked against the accepted range (section A). If there are no errors, the sensor level for the titration endpoint is set (section C). For the LDR light sensor case, during the titration process, the sensor value increases. Thus, the endpoint selected must be a greater value than that of the starting point (this requirement is checked by the software). For this purpose, two potentiometers (R4 and R5 shown in Figure 1) were operated. The plot in the screenshot (Figure 4) shows the evolution of the detector signal during the progress of the simulated titration. For these dummy tests, serial communication was not inhibited, allowing for various stages to be plotted, which are labeled by the numbers 1, 2, and 3 in section D. The horizontal red line indicates the limit value of the endpoint sensor. Further details on the measuring operation can be found in the Supplementary Materials.

The complete program code, with remarks, can be supplied by the authors upon request.

## 3. Results and Discussion

In this section, the main results obtained when using true electrochemical cell tests will be presented. The issues encountered and possible improvements are also analyzed and discussed. Two common coulometric reagents were electro-generated in various combinations, assemblies, and different configurations [36]. All measurements were performed at a controlled temperature of 25 ± 2 °C.

Before using the galvanostat, it is important to have the current calibrations for the different current scales available. Precision resistors are connected at the electrode terminals for the different scales. The system exhibits a monotonic and perfectly linear relationship between the measured current and the PWM digital value, with less than 1% error, independently of the selected scale. Considering the current scale used (sensitivity), a single linear regression (slope, intercept) can also be used for all scales (see the Supplementary Materials for a detailed description of the calibration process).

### 3.1. Iodometry Analysis

The main redox reaction in iodometric titrations is the reduction of iodine to iodide and, conversely, the oxidation of iodide to iodine:

$$I_{2}\left(aq\right)+2e^{-}⇄2I^{-}\left(aq\right)$$

**Figure 5:**
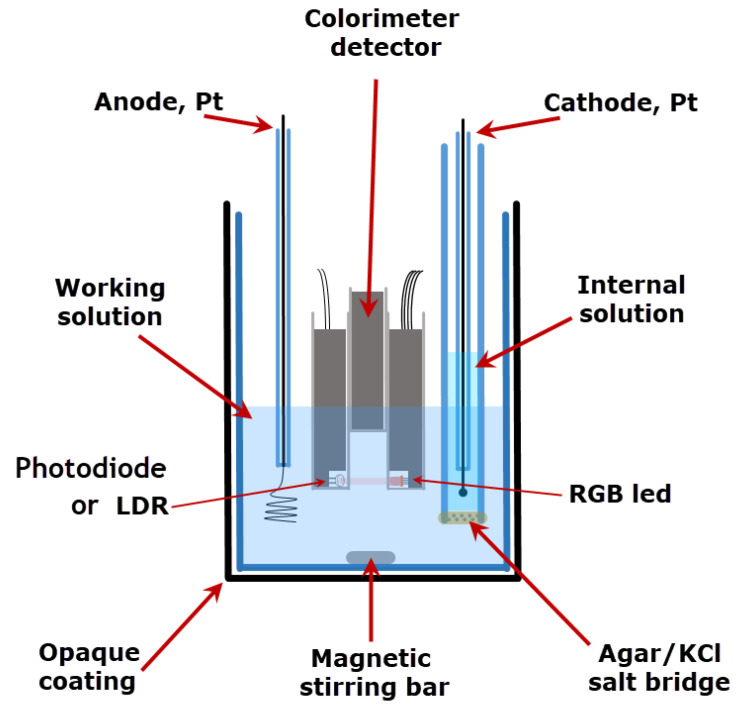
Schematics of the two-compartment coulometric cell employed for iodometric titrations.

Thus, iodine is generated at the anode by oxidation of the iodide, but the iodine produced can be reduced again at the cathode. Therefore, the coulometric electrolytic cell needs to have separate compartments, as can be seen in Figure 5. A glass tube containing fritted glass at the bottom with a layer of agar–agar filled with a saturated KCl solution, was used as a salt bridge. In this way, convective reactant transport from one compartment to another was hindered, keeping the electrical resistivity of the membrane low.

Molecular iodine is not soluble in water, but is solubilized in the presence of iodide, forming iodine–iodide complexes such as $I_{3}^{-}$ or $I_{5}^{-}$. These anions intercalate into the starch structure, forming a charge transfer complex with an intense blue color, with a broad absorption band in the visible range between 600 nm and 650 nm [37]. A few drops of colloidal starch solution added to an iodide solution will cause the solution to turn a blue color in the presence of iodine. The light emitter endpoint detector is illuminated by the red light of the RGB LED, thus obtaining optimal sensitivity.

Two types of iodometric titrations were performed using *I*_2_ as the oxidizing agent: the classical thiosulfate titration is according to the reaction:

$$2S_{2}O_{3}^{2-}\left(aq\right)+I_{2}\to S_{4}O_{6}^{2-}\left(aq\right)+2I^{-}\left(aq\right)$$

Subsequently, the titration of ascorbic acid is according to the reaction:

$$C_{6}H_{8}O_{6}\left(aq\right)+I_{2}\to C_{6}H_{6}O_{6}\left(aq\right)+2I^{-}\left(aq\right)+2H^{+}\left(aq\right)$$

Thus, taking into account the stoichiometry of these redox reactions, one Faraday is needed to oxidize one mole of thiosulfate ion, but two Faradays must be supplied for each mole of ascorbic acid.

The detector is sensitive to ambient light changes, so it should be insulated from sudden changes (partly cloudy day, fluorescent lights,…), hence the vessel was enclosed by an opaque casing. Furthermore, in these kinds of measurements, it has been observed that sudden and random changes in the detector can occur, even when shielded from ambient light. A careful inspection revealed that the colloidal starch solution formed highly colored particles that were visible to the naked eye and were sensed by the light detector. This issue was eliminated by modifying the software, so that the titration was carried out in several stages, as above-mentioned.

The following table summarizes the results estimated and obtained for the titration of 200 μL of the 0.07 M thiosulfate solution, 14 μmol, and for 100 μL of 0.05 M solution of ascorbic acid, 5.0 μmol. Table 1 shows that the error associated with the reproducibility (precision) of the measurements was around 1% for both types of titration.

In the case of thiosulfate titrations, the relative error with respect to the real value (accuracy) was somewhat higher than 3% by excess. However, in the case of ascorbic acid titrations, the relative error was around 2% by defect. The determination of ascorbic acid produces an error by defect due to the low stability of its aqueous solutions containing dissolved air [38].

### 3.2. Acid/Base Titrations

Another classic titration in teaching laboratories is the determination of the concentration of an acid by titration with hydroxide, using phenolphthalein as acid/base indicator. The appearance of a pink color is indicative of the endpoint of the titration.

If two Pt electrodes are employed for the electrolysis, OH- anions and hydrogen gas are generated by water reduction at the cathode: $2H_{2}O+2e^{-}\to H_{2}\left(g\right)+2{OH}^{-}(aq)$, while at the anode, the oxidation of water produces protons and oxygen gas:

$$2H_{2}O\to O_{2}\left(g\right)+4H^{+}\left(aq\right)+4e^{-}$$

Obviously, a two-compartment coulometric cell, analogous to the one shown in Figure 5, has to be used. However, there is a smart solution that uses a cell with just one compartment. For this purpose, a Pt cathode and Ag anode electrode are employed immersed in a single KBr 0.1 M solution [28]. The Ag^+^ ions produced at the anode react with Br^-^ and precipitate on the electrode as AgBr, and the formation of protons is inhibited.

**Figure 6:**
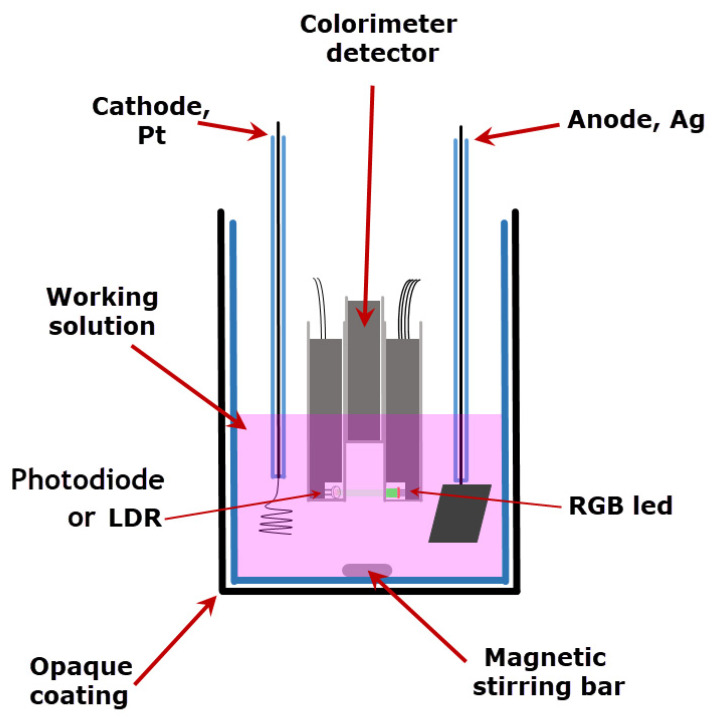
Schematics for a single-compartment coulometric cell employed for acid/base titrations.

A schematic of a one-compartment cell used in the titration experiment is depicted in Figure 6. In this case, as the phenolphthalein indicator has an absorption maximum at 550 nm, the emitter green LED was employed to improve the sensitivity.

The following table shows a comparative summary of the initial results obtained for the titration of a 5 mM HClO_4_ solution, using the two-compartment and one-compartment cell, for an added volume of 150 μL in both cases.

From Table 2, no differences in results were observed for the use of the two configurations of the electrochemical cells, with one or two compartments. For the sake of simplicity, the use of single-compartment cells, which were used in the subsequent acid/base titrations, are recommended. 

In these titrations, values in excess of the added amounts were always found, which may be an indication of the presence of CO_2_ dissolved by contact with air. As a result, excess amounts of acid were obtained, compromising the accuracy of the determinations.

However, it would be desirable to further reduce the systematic error in the titrations so that the system, in addition to serving educational purposes, can be used in other research areas. For this purpose, the LDR light detector was changed to a photodiode [32] to obtain better accuracy and sensitivity. By using the photodiode (OPT101 circuit), no rough/fine-tuning circuitry is needed for signal accommodation, as the voltage response provided can be read directly by Arduino’s analog converter.

This light detector has some advantages: linearity, fast response, and sensitivity. Nevertheless, there are some drawbacks, mainly due to its high sensitivity. Unstable external light should be avoided, so the electrolytic cell needs to be operated under dark conditions as colorimeters or spectrophotometers.

An inspection of the signal at the light detector showed a small dependence on the brightness of the emitting LED. An analysis of the PWM signal used to turn on the RGB LED showed a certain level of oscillation, imperceptible to the naked eye, due to PWM signal generation [29]. These oscillations were more evident using the photodiode as a detector. To minimize this noise, an active low-pass filter was inserted between the PWM signal and LED supply [39]. This active filter had the same configuration as the one used in the DC bias circuit used to apply the current (see Figure 3 in the Supplementary Materials in [23]; for more details on its connection, see the Supplementary Materials).

Furthermore, the stirring frequency of the electrolysis solution provided additional random noise. The light from the emitter was reflected by the stirrer bar and this light change was detected. This effect was minimized by using a very small stirring bar (1.5 cm × 2 mm diameter) and reducing the reflected light in the detector by blocking it with the Ag electrode (see Supplementary Materials for a description of noise sources and their correction and/or minimization).

**Figure 7:**
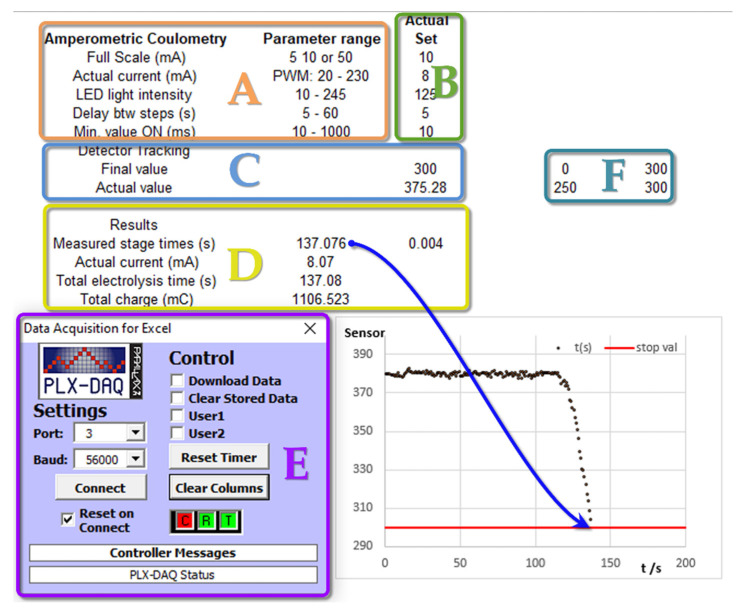
Section of an Excel screenshot for an acid/base coulometric titration using a photodiode OPT101 light sensor. (**A**) Input parameters and verification ranges. (**B**) Actual value set. (**C**) Detector levels set. (**D**) Measurement results. (**E**) Add-on module for serial communication. (**F**) Plot scales.

The best detector response was obtained by selecting the initial state of the solution without any color having been developed by the indicator compound. As the detector sensitivity is quite high, it is not recommended to use very high or very low RGB LED brightness values. A change of approximately 10% of the full analog scale, for the overall titration process, is adequate for accurate endpoint detection. Figure 7 shows a screenshot of a successful coulometric acid/base titration using the photodiode OPT101 as a light detector, with the above-mentioned improvements to increase the accuracy. Sections A–F have the same meaning, as indicated previously in Figure 4. As previously mentioned, the titration procedure has the same sequence to that explained for the dummy experiments. As the OPT101 device was employed, during the titration process, the sensor value decreased. Thus, the endpoint value had to be set lower than the starting point (section D). For the complete titration process, a change in the sensor value of about 100 units is recommended. The detector level is set by adjusting the brightness of the RDB led. In this case, there was no dummy titration and the plotting for the experimental results was only allowed for the first stage. For further details and recommendations, see the Supplementary Materials.

It was observed that the variation in the signal during the process of titration was the opposite of that obtained using the LDR, as the voltage developed by the photodiode decreased as the concentration of colored phenolphthalein increased. The change in the value of the detector signal, to reach the endpoint, had been amplified by five. Nevertheless, the base noise level remained relatively low. It is also noteworthy that the PWM value used to turn on the LED was considerably higher. The output filtered signal coming from the PWM pin, for small values, did not produce enough voltage to turn ON the RGB LED.

With all of these improvements, carefully controlled acid–base titrations were performed. Primary standard solutions of potassium hydrogen phthalate 0.05 M solutions were prepared by weighing with 0.1 mg precision, 5 µmol/0.1 mL (483 mC/0.1 mL). Micropipettes were re-calibrated by weighing distilled water. A total of 30 mL of 1 M NaBr was employed as the electrolytic solution. The summary results are presented in Table 3.

It should be noted that all measurements had an acceptable accuracy of below 1% RDS in the determination of micromole scale quantities. However, an excess error of around 3% in accuracy was still observed. This fact was found to be due to the presence of atmospheric CO_2_. An increase in the stirring frequency and/or the extent of titration increased this absolute excess error. This interference was also observed in manual titrations of acids with phenolphthalein, where the pink color disappeared several seconds after completion of the titration, which has been addressed in [8,40,41]. The elimination and study of this particular interference at the detection levels employed was beyond the scope of this paper. 

However, it should be noted that the proposed device provides reproducibility at very acceptable levels. It is possible to determine sub-micromolar quantities with an accuracy of about 0.5%.

## 4. Conclusions

The design and evaluation of the coulometric titrator with digitally controlled photometric endpoint detection consists of three basic components: a digital galvanostat, a photometric detector, and a microcontroller. This hardware ensemble was readapted by slightly modifying the original galvanostat design that included a simple photometric detector (emitter/receiver) and developing a new software code for the microcontroller. 

The whole system was developed with open source or free tools, with a total cost that did not exceed $200. The use of the microcontroller coupled to a data sheet (by means of a simple add-on) allowed for greater versatility in the system. The titration process was controlled directly from the spreadsheet. Therefore, the monitoring and checking of the multiple variables that control the experiment can be updated without having to modify the code stored in the memory of the microcontroller. This fact allows for multiple experiments with different conditions to be performed very quickly.

In addition, the new photometric detector also added an extra versatility, allowing experiments to be performed in the entire visible light spectrum, although its highest sensitivity was obtained when using one of the single RGB colors. Furthermore, the study and remediation of the experimental noise in the true electrolytic cells have made it possible to achieve results with an accuracy better than 1%. For this purpose, the use of additional active filters is required as well as the adaptation of the titration environment. 

Finally, it is worth emphasizing the great versatility of the equipment as a whole, which could be readapted relatively easily at very low cost in other types of experiments in which a precise time control and/or applied current is required.

## Figures and Tables

**Table 1:** A summary of the results for the iodometric titrations by using the two-compartment cell.

Thiosulfate					
# of Samples	n /μmol	i /mA	t Estimated /s	t Average /s	%RSD
16	14.	17.4	77.8	80.6	1.0
**Ascorbic acid**					
20	5.0	5.24	184	181	0.4

**Table 2:** Summary of the initial acid/base titrations.

Two-Compartment Cell					
Sample	n /μmol	i /mA	t Estimated /s	t Average /s	RSD %
15	0.75	5.23	13.8	15.4	3.2
**Single-Compartment Cell**					
15	0.75	5.24	13.8	15.3	3.9

**Table 3:** The results for standard potassium acid phthalate 0.05 M titrations.

Single-Compartment Cell					
# Sample	n /μmol	i /mA	t Estimated /s	t Average /s	% RDS
4	10.0	8.07	119.6	123.5	0.1
5	10.0	4.04	239.4	245.5	0.4
5	9.71	8.07	116.1	118.4	0.6

## Data Availability

Not applicable.

## Supplementary Materials

The following are available online at https://www.mdpi.com/article/10.3390/s22197541/s1, Figure S1. Screenshot of the PLX-DAQ add-on, showing the boxes to be unchecked once all pa-rameters are correct and the light sensor has an appropriate value; Figure S2. Unified plot for the different current scales, SE, a single linear relationship can be used for each scale, 5, 10 and 50 mA. Figure S3. Schematic of the connections for an active filter Butterworth two poles. Cut-off fre-quency is calculated as: $f_{c}=\frac{1}{2\pi \sqrt{C_{1}\cdot C_{2}\cdot R_{1}\cdot R_{2}}};\;C_{1}/C_{2}≃2$. Figure S4. Snapshots of several frames of a video taken during an acid/base titration. Small H2 bubbles are clearly observed moving and modifying the light signal reaching the photodiode. Figure S5. Detector signal of photodiode at the start of a titration coupled (aliased), with the flickering (100 Hz) of lab fluorescent light and the plotting acquisition period. Figure S6. Change of the detector photodiode signal for a titration with an unattainable end-point. The detector signal starts with a nearly constant value (color is not yet developed), then the indicator color appears and the signal drops asymptotically to a new value. The zone marked in blue corresponds to the more abrupt signal change. Figure S7. Variation of the absorbance change of the detector photodiode signal during a titration. The points marked in blue correspond to the most rapid change, op-timal for endpoint selection.

## References

[1] Ewing G.W. (1985). Instrumental Methods of Chemical Analysis.

[2] Chemistry Libretexts: Coulometric Methods. https://chem.libretexts.org/Bookshelves/Analytical_Chemistry/Analytical_Chemistry_2.1_(Harvey)/11%3A_Electrochemical_Methods/11.03%3A_Coulometric_Methods.

[3] Swift E.H. (1956). Coulometry. Anal. Chem..

[4] Bard A.J. (1966). Electroanalysis and Coulometric Analysis. Anal. Chem..

[5] Bard A.J., Faulkner L.R. (2000). Electrochemical Methods, Fundamentals and Applications.

[6] Bakker E. (2016). Can calibration-free sensors be realized?. ACS Sens..

[7] Harris S., Gonzales J., Melaku S., Dabke R.B. (2019). Feasibility of performing concurrent coulometric titrations using a multicompartment electrolysis cell. ACS Omega.

[8] Recknagel S., Breitenbach M., Pautz J., Lück D. (2017). Purity of potassium hydrogen phthalate, determination with precision coulometric and volumetric titration—A comparison. Anal. Chim. Acta.

[9] Bing W., Qiyue C., Pengfei X. (2013). Determination purity of benzoic acid by high precision constant current coulometry. Xiandai Yiqi Yu Yiliao (Mod. Instrum.).

[10] Padilla Mercado J.B., Konkolewicz D., Bretz S.L., Danielson N.D. (2017). Indirect determination of zinc by thiol complexation and iodine coulometric titration with photocell detection. Microchem. J..

[11] Ziyatdinova G., Ziganshina E., Budnikov H. (2012). Surfactant media for constant-current coulometry. Application for the determination of antioxidants in pharmaceuticals. Anal. Chim. Acta.

[12] Kong Q., Wu J., Chen M., Chen Z. (2022). Coulometric back titration based on all-soli-stated electrodes for phenylephrine hydrochloride determination. Anal. Bioanal. Chem..

[13] Talebi M., Amstrong D.W., Riley C.M., Rosanske T.W., Reid G. (2020). Water determination (Chapter 17). Specification of Drug Substances and Products: Development and Validation of Analytical Methods.

[14] Siano F., Picariello G., Castaldo D., Cautela D., Caruso T., Vasca E. (2022). Monitoring antioxidants by coulometry: Quantitative assessment of the strikingly high antioxidant capacity of bergamot (*Citrus bergamia* R.) by-products. Talanta.

[15] Ziyatdinova G., Budnikov H. (2015). Electroanalysis of antioxidants in pharmaceutical dosage forms: State-of-the-art and perspectives. Monatsh. Chem..

[16] Suarez H., Cristancho R., Peralta F., Torres H. (2017). Implementation of coulometric titration system at constant current for developing of certified materials as primary standards. J. Phys. Conf. Ser..

[17] Suiter C.L., Widegren J.A. (2021). Hygroscopic tendencies of substances used as calibrants for quantitative nuclear magnetic resonance spectroscopy. Anal. Chem..

[18] Sandstorm D.J., Offord B.W. (2022). Measurement of oxygen consumption in Tenebrio molitor using a sensitive, inexpensive, sensor-based coulometric microrespirometer. J. Exp. Biol..

[19] Padilla Mercado J.B., Coombs E.M., De Jesus J.P., Bretz S.L., Danielson N.D. (2018). Iodine Coulometry of Various Reducing Agents Including Thiols With Online Photocell Detection Coupled to a Multifunctional Chemical Analysis Station To Eliminate Student Endpoint by Eye. J. Chem. Educ..

[20] Eivind J.A., Kvittingen V., Kvittingen L., Verley R. (2014). A Simple, Small-Scale Lego Colorimeter with a Light-Emitting Diode (LED) Used as Detector. J. Chem. Educ..

[21] Dabke R.B., Gebeyehu Z., Thor R. (2011). Coulometric analysis experiment for the undergraduate chemistry laboratory. J. Chem. Educ..

[22] Jeffery G.H., Bassett J., Mendham J., Denney R.C. (1989). Vogel’s Textbook of Quantitative Chemical Analysis.

[23] González-Arjona D., Roldán E., López-Pérez G., Domínguez M.M. (2012). Versatile Instrumental Assemblage for the Study of Commercial Electrochemical Cells. Chem. Educ..

[24] Arduino Microcontrollers. https://www.arduino.cc/.

[25] Mabbott G.A. (2014). Teaching Electronics and Laboratory Automation Using Microcontroller Boards, 2014. J. Chem. Educ..

[26] Parallax Data Acquisition Microcontroller Tool. https://www.parallax.com/package/plx-daq/.

[27] González-Arjona D., Roldán González E., López-Pérez G., Domínguez Pérez M.M. (2013). An Improved Galvanostat for the Characterization of Commercial Electrochemical Cells. J. Lab. Chem. Educ..

[28] Lötz A. (1998). A Variety of Electrochemical Methods in a Coulometric Titration Experiment. J. Chem. Educ..

[29] Hirzel T., Arduino Basics of PWM (Pulse Width Modulation). https://docs.arduino.cc/learn/microcontrollers/analog-output.

[30] RS Online, 5050 RGB LED. https://docs.rs-online.com/4079/0900766b813cbc6a.pdf.

[31] Token Electronics LDR Photoresistor PGM5. http://www.token.com.tw/resistor/photo-cds.htm?id=light-sensor?_3.

[32] Texas Instrument. OPT101 Light to Voltage Converter Data Sheet. https://www.ti.com/product/OPT101?keyMatch=OPT101.

[33] Stack Overflow Company Electrical Engineering: Circuit of a Coarse and Fine Setting Potentiometer. https://electronics.stackexchange.com/questions/144530/circuit-for-a-coarse-and-fine-setting-potentiometer.

[34] Domínguez Pérez M.M., Roldán González E., López-Pérez G., González-Arjona D. (2020). BatTest Galvanostat. A Digitally Controlled-Current Instrument. Building-Up BatTest 19. https://www.researchgate.net/publication/339210809_Building-up_BatTest_19.

[35] Christian G.D. (1965). Coulometric Titration of Hydrogen Peroxide with Electrogenerated Iodin. Anal. Chem..

[36] Kanyaneeab T., Fuekhada P., Grudpanab K. (2013). Micro coulometric titration in a liquid drop. Talanta.

[37] Yu X., Houtman C., Atalla R.H. (1996). The Complex of Amylose and Iodine. Carbohydr. Res..

[38] Silva R.C., Simoni J.A., Collins C.H., Volpe P.L.O. (1999). Ascorbic Acid as a Standard for Iodometric Titrations. An Analytical Experiment for General Chemistry. J. Chem. Educ..

[39] Scherz P., Monk S. (2016). Practical Electronics for Inventors.

[40] McAlpine R.K. (1944). The Carbon Dioxide Problem in Neutralization Titrations. J. Chem. Educ..

[41] Stelmach E., Maksymiuk K., Michalska A. (2016). Copolymeric hexyl acrylate-methacrylic acid microspheres—Surface vs. bulk reactive carboxyl groups. Coulometric and colorimetric determination and analytical applications for heterogeneous microtitration. Talanta.

