# Supplementary material for: Coulometer from a Digitally Controlled Galvanostat with Photometric Endpoint Detection
Source: Sensors (Basel). 2022 Oct 5;22(19):7541. doi: 10.3390/s22197541 (PMC9570983; doi:10.3390/s22197541)
Supplement: Supplementary file 1 [file sensors-22-07541-s001.zip › sensors-1930412-supplementary.pdf]

Supplementary material

Coulometer with Photometric End-Point Detection, from a Digitally Controlled Galvanostat

Summary

The following supplementary material is provided for clarifications and details for the following aspects mentioned in the original paper:

- Software description: validation of parameters, issues with RS232 communication, saving memory recourses, routines description, adaptation of light detector.
- Galvanostat calibration procedure.
- Additional hardware: Filters for noise minimization of different sources.
- Measurement process: noise sources detection and minimization, optimal configuration for better accuracy.

Software description.

The general structure of the Arduino controller programs contains a cyclically repeating section, the void loop(). However, the titration procedure consists of a single process and therefore the code included in that section is executed only once. The use of the interface with the Excel add-on, allows control of the different ranges of parameters of the titration, avoiding having to upload them to the program each time one or a set of parameters is changed [15, 16]. The numbering of the bibliographic references corresponds to those cited in the article.

The parameter validation process is a loop in which the values are sequentially checked. Once there are no errors, the program proceeds to the selection and adjustment of the value for the endpoint of the titration. It is necessary to select a higher value than the initial value when using the LDR sensor or a smaller value when the photodiode sensor is employed. Once the endpoint value fulfills the conditions, it is accepted. Then, two checkboxes of the add-on need to be unchecked for the program to continue, see Figure S1.

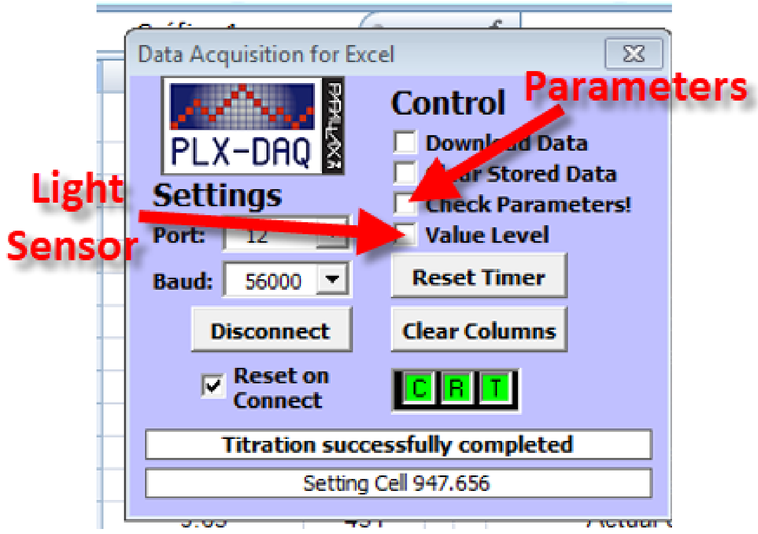

Figure S1. Screenshot of the PLX-DAQ add-on, showing the boxes to be unchecked once all parameters are correct and the light sensor has an appropriate value.

In this way the desynchronization between the read/write via RS232 and the read/write performed on the spreadsheet is avoided. However, sometimes desynchronizations occur and it is necessary to disconnect and reconnect the add-on via its button.

The number of variables in the program is relatively large for an Arduino UNO which has a 2kB SRAM memory for variable manipulation. Therefore, part of the text messages sent to the Arduino have been stored in the Flash memory where the compiled program resides, 32k for the Arduino UNO. For this, the "F" command is used to print character strings or informational text, for example: Serial.print(**F**("MSG,Stage "));

However, other compatible and higher-performance microcontrollers in the family, such as the MEGA 2560 or the Arduino Zero [13], can be used.

Subroutines.

void Check_inPin( ) // Status detection for manual emergency stop.

void Check_analogValue() // Checks the voltage between the electrodes. If the value is higher than a preset value, this indicates a high resistance between the electrodes.

void Promedio () // Average of 10 light sensor measurements.

float Promedio_Exp(float avg, float new_sample, int number) // Moving average routine.

void Diff_Float_Promedio () // Averaging of the signal from two light detectors, one in the solution and the other external. Not used in the project.

void Medida () // Subroutine in which the stages of the measurement process are performed. For the first stage, the graphical representation is performed simultaneously.

if (indice == **0**) //Only plot for first stage, avoiding timewasting.

The type of sensor to be used, LDR or photodiode, requires prior configuration, commenting the appropriate lines of code. So that the endpoint can be detected.

//Version Photo-transistor getting darker, decrease voltage
while ((punto_final_actual **>** punto_final) && (digitalRead(inPin2) == LOW) && (analogValue <= voltaje_error));

//Version LDR getting darker, increase voltage
while ((punto_final_actual **<=** punto_final) && (digitalRead(inPin2) == LOW) && (analogValue <= voltaje_error));

void Setup_head() // Writing of the text explaining the titration parameters.

void calibrado_sensibilidad() // Selection of the values of the slope and ordinate at the origin according to the value of the sensitivity, SE, of the selected current. Using individualized equations for each sensitivity is possible:

current_mA = float(i_FE * PWM / 255.0) * slope + intercept;

The slope and intercept values for each sensitivity scale are obtained separately by the calibration process, and have to be included in the code

void GAL_ON () and void GAL_OFF () // set galvanostat ON/OFF

The developed program is available from the authors upon request.

Galvanostat calibration procedure.

A low impedance resistor, 100 Ω with low temperature coefficient is used as dummy cell.

A precision ammeter is used to measure the actual current. Some PWM values are set along the selected current scale and the linear correlation between them is performed. The individual linear parameters of each scale can be used.

However, a single linear correlation can be used for all scales by plotting the actual scaled current for each sensitivity, i_act/SE, against the PWM value. In this way with only the two parameters of the linear correlation the value of the current for the different scales has to be provided, saving some memory, and obtaining a cleaner programming code.

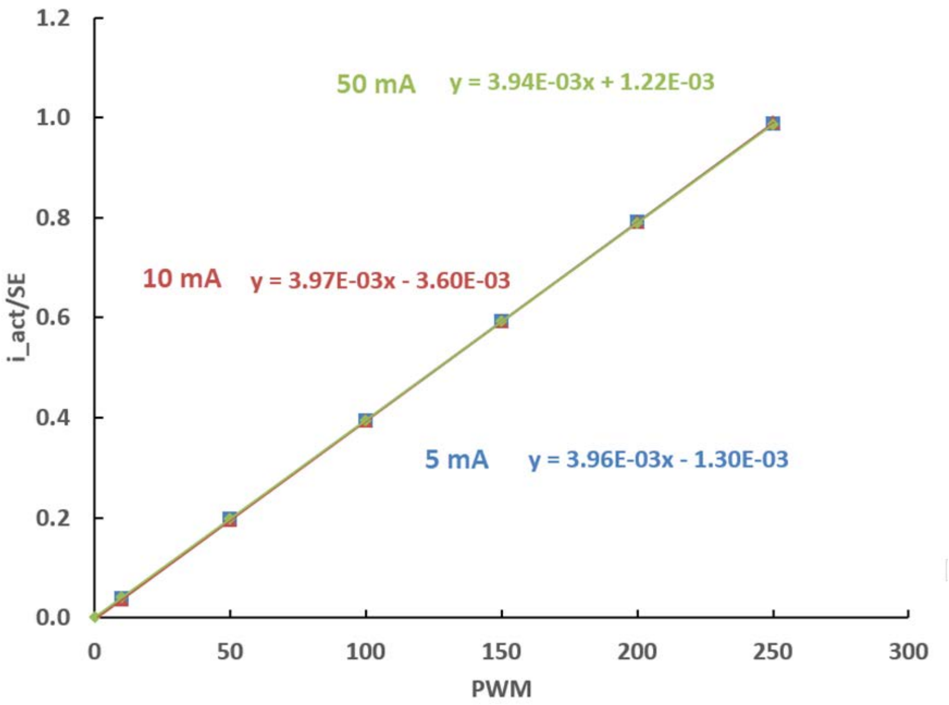

Figure S2. - Unified plot for the different current scales, SE, a single linear relationship can be used for each scale, 5, 10 and 50 mA.

For the prototype the average equation is:

i_act = (0.00396*PWM+0.001)*SE

A variable relative error is obtained due to the digital character of the PWM, which is an integer. Thus, when estimating the PWM value to use for a real current value, a floating value is obtained, which is truncated. However, the maximum relative error is always less than 0.3%. Figure S2 shows the results obtained for the three scales employed in the project (5, 10 and 50 mA).

Additional hardware: Filters for minimizing noise.

The interconnection between a digital device and an analog sensor creates certain difficulties in relation to the appearance of noise in the sensor signal. It is essential to minimize them by determining their origin and the use of filters serves to improve the signal to noise ratio without compromising the signal of interest. In the present case, the signal of interest can be considered to be of very low frequency, since it is modified as the electrolysis is performed, governed by the value of the electric current. Therefore, it is possible to use in the analog inputs low pass filters of RC type with relatively low cutoff frequency, in our case, 15Hz.

On the other hand, the light sensor with LDR has the signal conditioned by a voltage divider that is powered by a 5V source. This power supply could be provided by the microcontroller itself or even by the PC through its USB interface. Our solution, in order not to compromise the load of the digital equipment mentioned above, has been to employ an external low noise 5V power supply, less than 5mV peak to peak.

On the other hand, as discussed in the article, the illumination of the RGB LED is done from a PWM type signal. Most of the PWM type pins on the Arduino have a base frequency of approximately 500 Hz. The digital value can be considered as an analog value when using slow or insensitive response sensing devices, for example the human eye or an LDR. However, with the implementation of an amplified, more sensitive and faster responding device such as the OPT101, that base frequency is detected as random noise. To minimize this, a two-pole Butterworth-type active low-pass filter using an OA

AD711, has been included in the system, Figure S3. The values used for a cutoff frequency of 1Hz are R1=R2=1M, C1 and C2, 225 nF and 113 nF, respectively.

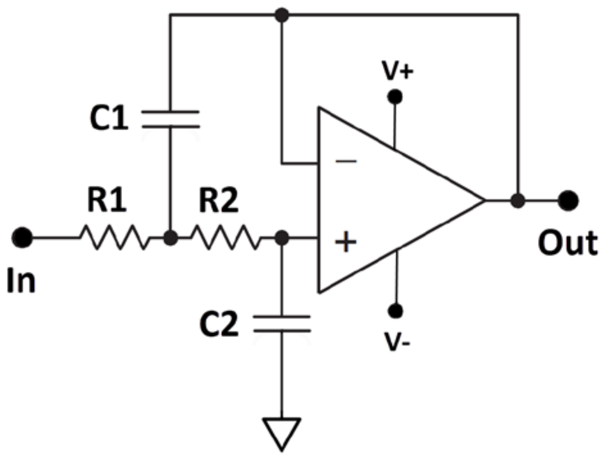

Figure S3. - Schematic of the connections for an active filter Butterworth two poles. Cut-off frequency is calculated as: $f_c = \frac{1}{2\pi\sqrt{C_1 \cdot C_2 \cdot R_1 \cdot R_2}}$; $C_1/C_2 \simeq 2$.

The implementation of this filter was done on a breadboard using the symmetrical power supply of the galvanostat itself. The input was connected to the PWM pin and the output to the power supply of the corresponding LED.

Apart from these noise sources, additional random noises have been found due to inhomogeneities in the electrolysis solution and the intermittent reflection of light of the LED from the magnetic stirrer. This source of noise has been minimized by using a low-profile, 2 mm stirring bar and placing the flat Ag electrode as an anti-reflection screen on the side of the phototransistor. Additionally, during the addition of successive sample aliquots, the appearance of small hydrogen bubbles was observed in the acid/base titrations. Figure S4 shows samples of some frames from a video taken during the titration process. All these random noises were filtered by software averaging of the signal using the Promedio subroutines.

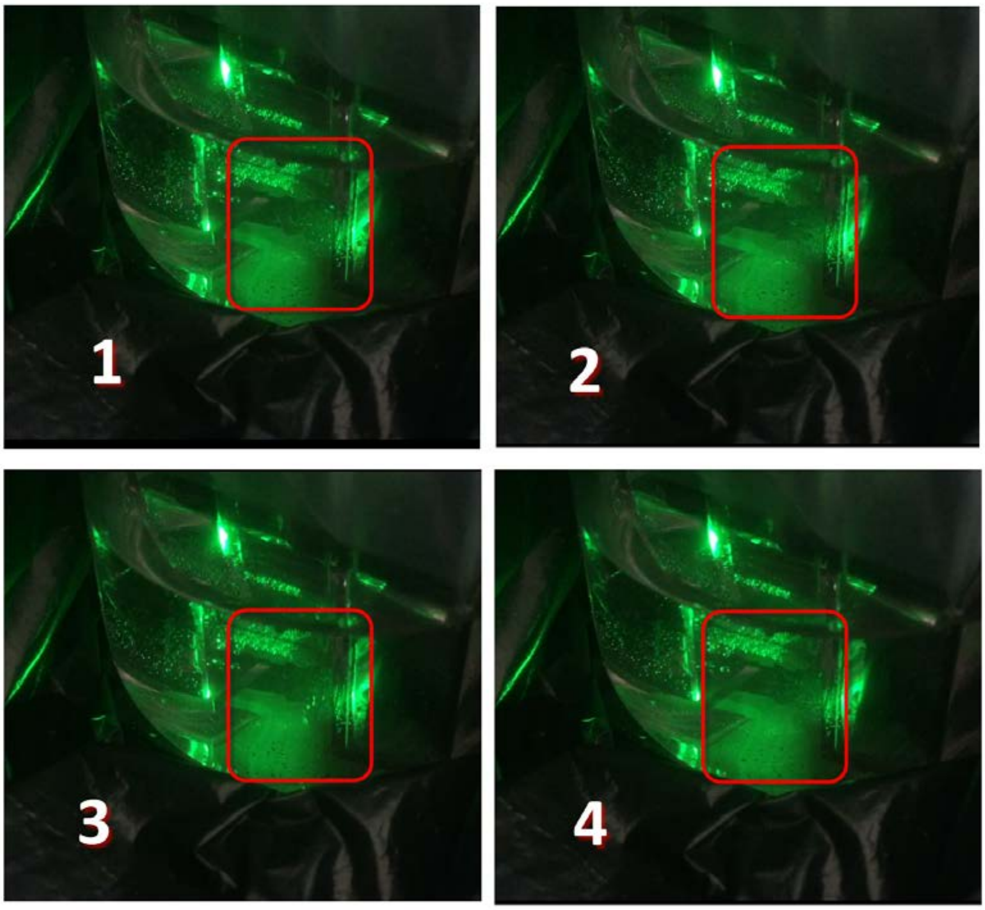

Figure S4. - Snapshots of several frames of a video taken during an acid/base titration. Small H_2 bubbles are clearly observed moving and modifying the light signal reaching the photodiode.

Isolation of artificial light sources is essential. The fluorescent light from the lab, which does not flicker sensibly to naked eye, couples with the signal received by the photodiode producing aliasing, a signal with a lower frequency than the original one. This fact was revealed by making measurements and observing the change in the titration signal, as shown in Figure S5.

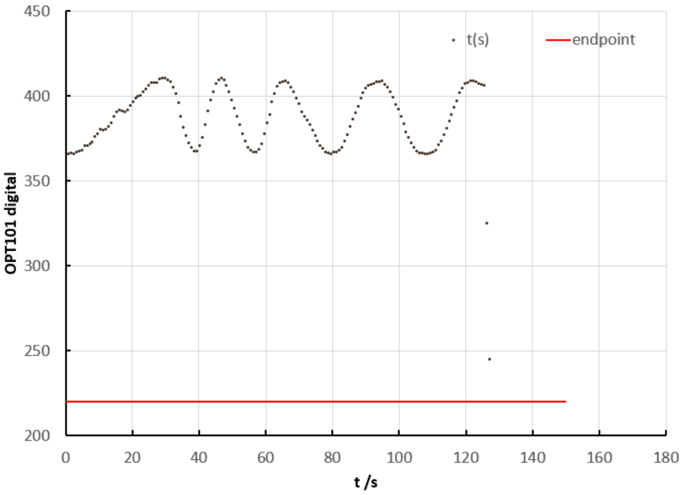

Figure S5. - Detector signal of photodiode at the start of a titration coupled (aliased), with the flickering (100 Hz) of lab fluorescent light and the plotting acquisition period.

Another important task is to determine the optimum value of the detector signal change during the titration. Figure S6 shows the variation of the light detector signal during the progress of a titration in which an unattainable endpoint was selected. A sigmoidal-type variation analogous to a logarithmic response can be observed.

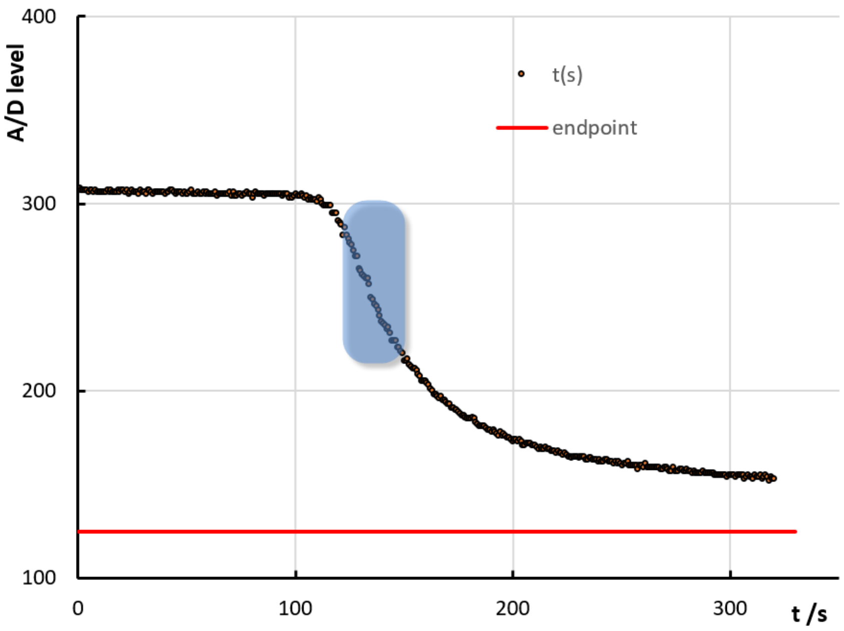

Figure S6. - Change of the detector photodiode signal for a titration with an unattainable endpoint. The detector signal starts with a nearly constant value (color is not yet developed), then the indicator color appears and the signal drops asymptotically to a new value. The zone marked in blue corresponds to the more abrupt signal change.

For best accuracy of the endpoint value, a zone of abrupt change of the detector signal should be chosen.

A logarithmic analysis of the detector signal level over time indicates that the optimum zone is in the region of linear change of the logarithm of the signal level, or absorbance, log(max_level/level). Figure S7 shows the latter variation, where the optimal zone of change has been marked. This corresponds to an approximate change in absorbance between 0.55 and 0.65. This in turn corresponds to a change of about 100 units in detector

levels, 10% of its full-scale range (0-1023).

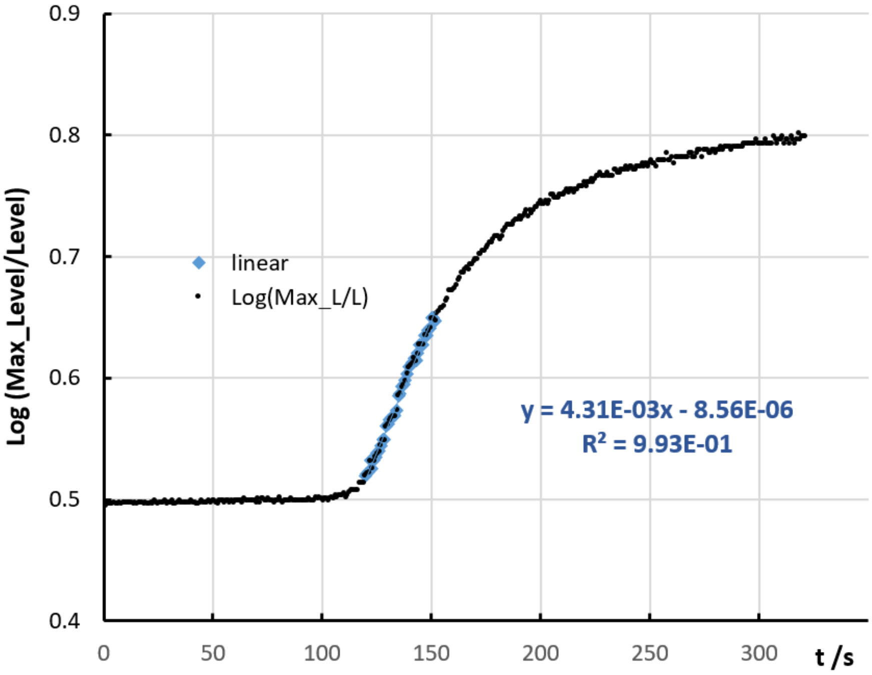

Figure S7. Variation of the absorbance change of the detector photodiode signal during a titration. The points marked in blue correspond to the most rapid change, optimal for endpoint selection.

An initial sensor level that is in the intermediate range of the analog-to-digital converter is recommended. Arduino UNO has a 10-bit A/D converter, using 5V as the full scale value, implies approximately 5mV for each step. Thus, for an initial value of 3V (approx. 600 converter units), selecting the final color value around 520 converter units, 2.6V, is advisable.
